# The many faces of Polycomb regulation by RNA

**DOI:** 10.1016/j.gde.2020.02.023

**Published:** 2020-04

**Authors:** Mafalda Almeida, Joseph S Bowness, Neil Brockdorff

**Affiliations:** Developmental Epigenetics, Department of Biochemistry, University of Oxford, Oxford OX1 3QU, United Kingdom

## Abstract

Many intricate pathways contribute to the timely control of gene expression during development. Polycomb repressive complexes (PRC1 and PRC2) and long non-coding RNAs (lncRNAs) are players associated with gene repression in various developmental processes such as X chromosome inactivation (XCI) and genomic imprinting. Historically, lncRNAs were proposed to directly recruit PRC2. However, recent evidence suggests that promiscuous interactions between PRC2 and RNA fine-tune the function of the complex through a multiplicity of mechanisms. A PRC2-recruitment model was definitively overturned in the paradigm of XCI by Xist RNA, being replaced by a novel mechanism which puts PRC1 in the spotlight. This review focuses on these recent advances in understanding the interplay between RNA and Polycomb complexes for gene expression control.

**Current Opinion in Genetics and Development** 2020, **61**:53–61

This review comes from a themed issue on **Genome architecture and expression**

Edited by **Kerstin Bystricky** and **Matthias Merkenschlager**

For a complete overview see the Issue and the Editorial

Available online 11th May 2020

**https://doi.org/10.1016/j.gde.2020.02.023**

0959-437X/© 2020 The Author(s). Published by Elsevier Ltd. This is an open access article under the CC BY license (http://creativecommons.org/licenses/by/4.0/).

## Introduction

The Polycomb family of genes was first identified in the 1980s in relation to their role in the maintenance of *Hox* gene silencing in specific segments of the *Drosophila* body plan during development (reviewed in Ref. [1]). Extensive biochemical and genetic experiments contributed to the identification and characterisation of Polycomb group (PcG) proteins in *Drosophila* as well as many homologues in mammals. PcG proteins form catalytically active complexes generally divided in two subgroups: Polycomb repressive complex 1 (PRC1) which catalyses H2AK119ub1, and Polycomb repressive complex 2 (PRC2) which methylates lysine 27 of histone H3 (H3K27me1/2/3) (reviewed in Ref. [2,3]). Besides its catalytic activity, PRC1 affects 3D chromatin structure in the nucleus (reviewed in Ref. [4]). Because of the dynamic nature of chromatin changes mediated by PRC1 and PRC2, timely regulation of Polycomb-mediated repression is fundamental to proper embryonic development.

**Figure 1:**
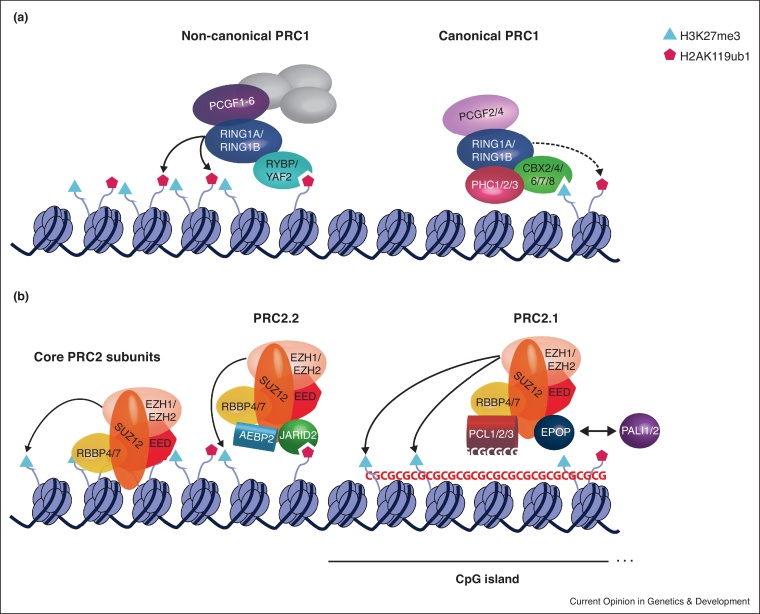
Polycomb repressive complexes subunits, recruitment mechanisms and catalytic activity in mammals. **(a)** PRC1 complexes are subdivided into canonical and non-canonical PRC1. Canonical PRC1 is characterised by the presence of one CBX protein (CBX2/4/6/7/8) that recognises H3K27me3 deposited by PRC2 and mediates recruitment of the complex and a PHC protein (PHC1/2/3), which dimerises and promotes chromatin folding. In this subtype of PRC1 complex, PCGF2 and PCGF4 bind in a mutually exclusive way to RING1A/B. Canonical PRC1 has residual catalytic activity (as represented by the dashed arrow). Non-canonical PRC1 complexes are the most catalytic active and deposit H2AK119ub1 at target genes (represented by full arrows). They are characterised by the presence of RYBP/YAF2 which are able to recognise the H2AK119ub1 mark and promote a positive feedback loop for reinforcement of non-canonical PRC1 recruitment. Non-canonical PRC1 complexes contain one of six PCGF proteins (PCGF1-6), some of which determine recruitment to specific targets due to interaction with other components like transcription factors (represented in grey). **(b)** PRC2 complexes are subdivided into PRC2.1 and PRC2.2 subtypes. Both subtypes share the same core components: EZH1/EZH2, the subunit that catalyses H3K27me3 deposition (as represented by the full arrows), EED which recognises H3K27me3 contributing to the propagation of PRC2 and reinforcement of its own mark (represented on the left), SUZ12, a vital structural component, and the subunit RBBP4/7. PRC2.1 includes the substoichiometric components PCL1/2/3, which are able to bind directly to CG-rich DNA and promote recruitment of this PRC2 subtype to target regions, and the mutually exclusive subunits EPOP and PALI1/2, which modulate its catalytic activity. PRC2.2 includes the substoichiometric components AEBP2 and JARID2, which also impact the catalytic activity of the complex. JARID2 is able to recognise the H2AK119ub1 mark deposited by PRC1 and this serves as a mechanism of recruitment for the PRC2.2 subtype.

The diversity of subunits of PRC1 and PRC2 present in mammals results in a wide variety of multimeric complexes (Figure 1) with consequences regarding structure, recruitment mechanisms, dynamics and function in the cell. ChIP-sequencing (ChIP-seq) experiments of Polycomb core components (RING1B and SUZ12) show their enrichment over CpG island promoters of developmentally regulated genes [5]. Given that unveiling the composition of all PcG protein complex variants is still a work in progress [2,3] and that marks of Polycomb activity (in this case, H2AK119ub1 and H3K27me1/2) are present outside of the complexes’ genomic targets identified by ChIP-seq [6,7], it has been challenging to establish a model to fully explain the intricacies of Polycomb recruitment genome-wide.

Much of the mammalian genome is transcribed into non-coding forms of RNA, such as long non-coding RNAs (lncRNAs) which have diverse cellular functions (reviewed in Ref. [8]). The correlation between the presence of the PRC2 mark — H3K27me3 — with the expression of lncRNAs with known functions in developmental processes, such as X chromosome inactivation [9] and genomic imprinting [10,11], led researchers to investigate binding to RNA as a mechanism of Polycomb recruitment. In this review we describe how the field has switched from the idea of direct PRC2 recruitment by RNA to a model by which RNA binding contributes to local fine-tuning of PRC2 function by multiple mechanisms. We emphasise a newly described mechanism that involves RNA-mediated PRC1 recruitment through an adaptor protein, hnRNPK, in the context of X chromosome inactivation by the lncRNA Xist as well as in RNA-mediated genomic imprinting.

## PRC2 interaction with RNA regulates its function rather than its recruitment

At the beginning of the century, increasing interest in the function of lncRNAs in parallel with an inability to fully explain Polycomb targeting to chromatin led several research groups to investigate if these two could somehow be linked. The hypothesis that lncRNAs could contribute to Polycomb recruitment seemed plausible since phenomena like X chromosome inactivation, which is dependent on the expression of lncRNA Xist [12], or genomic imprinting, which requires the expression of specific lncRNAs like Kcnq1ot1 *in cis* [13], were shown to be defective in mice lacking the core PRC2 component EED. The majority of early studies focused on demonstrating if these RNAs could directly recruit PRC2 to chromatin to promote gene silencing, reflecting the widely held view that PRC2 functions upstream of PRC1. These reports, together with work on HOTAIR RNA, a classical example of an RNA that controls transcription *in trans* [14], and many others (reviewed in Ref. [15]), made use of *in vitro* RNA binding assays and/or RNA-immunoprecipitation (RIP) assays to demonstrate apparently specific interactions between defined lncRNAs and PRC2 subunits (specifically, EZH2 [16,17], SUZ12 [18,19] and the substoichiometric subunit JARID2 [20]). A few examples of PRC1 direct binding to RNA mediated by CBX7 were also published [21,22] but these have not been extensively revisited [23] and its biological role remains unclear.

**Figure 2:**
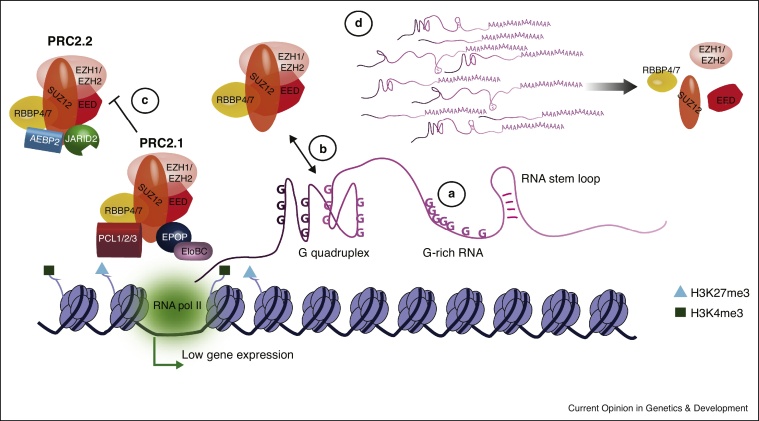
PRC2-RNA interaction depends on RNA structure and composition modulating PRC2 function. **(a)** PRC2 binds to RNA molecules promiscuously but its affinity varies with the sequence and folding of the RNA: G-quadruplex structures have the highest affinity for PRC2 binding, with unstructured G-rich RNA showing an intermediate binding affinity (contrary to A-rich RNA which binds minimally to PRC2) and RNA stem loops low affinity binding. **(b)** PRC2 binds preferentially to G-quadruplex containing RNAs and these are able to compete with PRC2 binding to chromatin, resulting in its displacement and reduced H3K27me3 deposition. RNA binding to PRC2 can also directly inhibit its catalytic activity. **(c)** PRC2.1 containing EPOP maintains a low level of gene expression at its target genes. Enrichment of EPOP-PRC2.1 and/or transcription keeps PRC2.2 further away from chromatin at these targets and prevents accumulation of H3K27me3 and complete gene silencing. **(d)** Accumulation of nuclear pA^+^ RNAs in the absence of efficient RNA degradation by a nuclear RNA exosome-mediated pathway leads to destabilisation of the PRC2 complex.

In the X inactivation field it was initially proposed that PRC2 is recruited by direct interaction with Xist/RepA [24,25^••^]. Subsequent observations challenged this (reviewed in Ref. [26]) and indeed an alternative mechanism has now been demonstrated (see below), yet the ability of PRC2 to bind RNA was confirmed in several later studies [17,19,20,27, 28, 29]. Resolution of this discrepancy has come from the recognition that PRC2 subunits interact promiscuously with RNA, including lncRNAs, short RNAs transcribed from the 5’ end of Polycomb target genes, and mRNA produced from active genes [29]. These observations deriving from EZH2 RIP-seq experiments were further corroborated by *in vitro* experiments that were central in showing that PRC2 binds to RNA promiscuously with different affinities depending on RNA length, sequence and structure [24,25^••^,^29^,30^••^]. PRC2 has high affinity for G-rich RNA, particularly when forming G-quadruplex structures (Figure 2), but it also has the ability to bind to other RNAs (like the stem loop structure of Xist/RepA) and even bacterial RNA [25^••^].

Several studies have recently explored the role of this promiscuous PRC2-RNA interaction. Importantly, these mechanisms are not mutually exclusive and probably cooperate to fine-tune PRC2 function in the cell.

RNA can inhibit the methyltransferase activity of PRC2 [31,32] by binding to a regulatory allosteric site in proximity to the catalytic centre [33^••^]. It is possible that RNA inhibition of the complex functions to prevent H3K27me3 deposition during chromatin scanning by PRC2, which may itself be facilitated by RNA binding [29]. RNA inhibition of PRC2 can be relieved by pre-existing H3K27me3 or JARID2 methylation, at least *in vitro* [33^••^], thus these may act as cues at specific sites leading to H3K27me3 deposition/propagation and gene silencing. Other mechanisms of PRC2 recruitment specific for each complex subtype, such as DNA binding by PRC2.1 [34] and H2AK119ub1 binding by PRC2.2 [35,36] (Figure 1), may also change the dynamics and local catalytic activity of the core PRC2 complex, but the ability of these to counteract RNA inhibition has not yet been tested.

Another observation is that RNA competes with DNA/chromatin for PRC2 binding [37,38], and in tethering experiments G-quadruplex-containing RNA displaces the complex leading to a local decrease of H3K27me3 without always reactivating transcription [30^••^]. In this situation, gene activation is context dependent [30^••^], therefore suggesting that PRC2 displacement from chromatin by RNA binding might provide the opportunity for changes in gene expression during development or disease.

Proper localisation of PRC2 complexes to target genes collectively requires PRC2 substoichiometric subunits [39,40]. It is plausible that this is affected by associations with RNA. For example, the PRC2.1 subunit EPOP interacts with Elongin BC to sustain low levels of expression at target genes, which counterbalances complete silencing by overaccumulation of PRC2.2 (Figure 2) [41,42]. Notably, EPOP is mutually exclusive with another PRC2.1 subunit, PALI1/2, and their respective knockouts yield opposite results regarding H3K27me3 deposition [42,43]. Thus, transcripts produced at EPOP-containing PRC2.1 sites may directly regulate this balance between PRC2.1 and PRC2.2 complexes on chromatin to result in different gene expression outcomes, although this has not been directly investigated.

More recently, two independent publications suggested that complexes involved in RNA processing and degradation, namely the rixosome (aka 5FMC complex) [44] and the nuclear RNA exosome [45^•^], can impact on Polycomb-mediated silencing through distinct mechanisms. In the latter, the authors found that disruption of the nuclear RNA exosome pathway leads to accumulation of pA^+^ nuclear RNA, resulting in impaired PRC2 binding to chromatin and destabilisation of the complex itself (Figure 2) [45^•^].

Taken together, recent evidence argues against the idea that RNA binding contributes to PRC2 recruitment. Instead, RNA binding to PRC2 fine tunes its activity, keeping PRC2 poised and in check as previously proposed [15,32]. This does not rule out that some lncRNAs might promote Polycomb recruitment to target genes, but this is most likely mediated by different mechanisms (see below).

## A new mechanism for RNA-mediated Polycomb recruitment in X chromosome inactivation and imprinted genomic regions

The recruitment of Polycomb complexes and their respective post-translational histone modifications are hallmarks of X chromosome inactivation (XCI) seen to occur rapidly in response to Xist RNA expression. Alongside other integral pathways [46^•^,^47^, ^48^, ^49^, ^50^], the interplay between Xist RNA and Polycomb is a key aspect of XCI in cellular and *in vivo* models and a paradigm for functional associations between Polycomb and lncRNA. After years of debate, there is an emerging consensus of how Xist RNA recruits Polycomb through a mechanism that seems to be common to other lncRNAs.

As noted above, early models for Xist-mediated Polycomb recruitment invoked a direct interaction of the PRC2 subunit EZH2 with the A-repeat element of Xist RNA [51], leading to PRC1 recruitment via the classical pathway of H3K27me3 recognition by CBX. Key findings that undermined this model were that Xist A-repeat deletion abolishes gene silencing but not Polycomb recruitment [52,53], and that PRC1 can be recruited by Xist in the absence of PRC2 [54,55]. Further studies applying RNA-pulldown proteomics [47,50] and super resolution microscopy [56] argued against a specific interaction between PRC2 and Xist RNA, and moreover found evidence for a closer association with non-canonical PRC1 subunits. The discovery of novel mechanisms of PRC2 recruitment at classical CpG island promoter sites also offered alternative pathways relevant in X inactivation [35,36].

**Figure 3:**
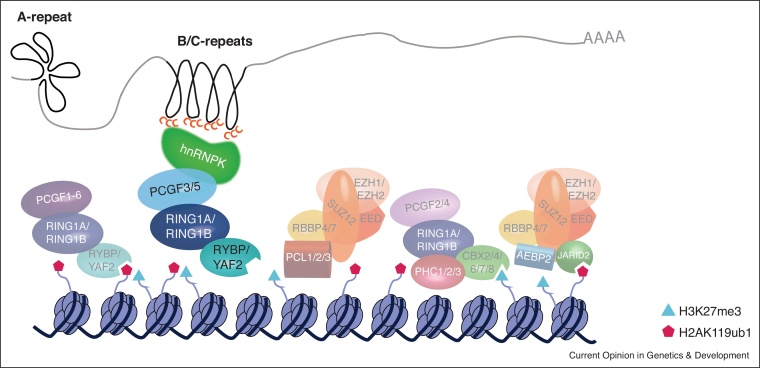
Model illustrating Polycomb recruitment by Xist lncRNA. Expression of the lncRNA Xist promotes enrichment of all subtypes of Polycomb complexes on the inactive X chromosome. This is mediated by the direct interaction between the B/C-repeat region of the RNA and a nuclear matrix protein, hnRNPK, which specifically engages PCGF3/5-PRC1 complexes. Downstream of initial PCGF3/5-PRC1 catalytic activity, self-reinforcing loops of recruitment acting through the recognition mechanisms represented in Figure 1 involve all non-canonical PRC1 complexes (via RYBP binding H2AK119ub1), PRC2 (via JARID2 binding H2AK119ub1) and canonical PRC1 (via CBX binding H3K27me3 deposited by PRC2).

A series of studies, using a variety of models and techniques (Table 1), have now revealed key details of the Polycomb recruitment pathway by Xist. The region of Xist RNA strictly required for recruitment of PRC1 and consequently PRC2 (see below) lies within the historical XN region [53,57^••^], notably distinct from the A-repeat, and encompasses a core ∼0.3 kb B-repeat sequence and the proximal C-repeat. This region is bound by hnRNPK [58^••^,59^•^,^60^], a nuclear-matrix associated protein with affinity for triplicate CCC-motifs [61] which are highly enriched within the B-repeat region (Figure 3). Loss of function experiments demonstrated a role for hnRNPK in Polycomb recruitment by Xist RNA [47,58^••^,59^•^].

**Table 1:** Recent studies converge upon B/C-repeats of Xist and hnRNPK as central players in Xist-dependent Polycomb recruitment

mESC, mouse embryonic stem cells; MEF, mouse embryonic fibroblasts; MS, mass spectrometry; ChIRP-MS, comprehensive identification of RNA-binding proteins by MS; LC, liquid chromatography; EMSA, electrophoretic mobility shift assay.

A landmark study showed a strict requirement for PRC1 upstream of PRC2 redefining the hierarchy of Polycomb complex recruitment in XCI and moreover defined a key role for the non-canonical PCFG3/5-PRC1 complex [57^••^]. The newly proposed model postulates that Xist/hnRNPK interacting with PCGF3/5-PRC1 is the first event of a cascade leading to Polycomb enrichment on the inactive X chromosome (Xi) [57^••^,58^••^]. PCGF3/5-PRC1 catalytic activity then establishes positive feedback loops involving recruitment of other non-canonical PRC1 complexes, PRC2 and consequently canonical PRC1 (Figure 3). If any of these downstream feedback pathways is ablated before Xist RNA induction Polycomb enrichment is reduced but not abolished [36,57^••^]. Recent studies employing ChIP-seq to map gain of Polycomb-associated histone modifications upon Xist expression have confirmed this model quantitatively [46^•^] and have shown that deposition of H2AK119ub1 dynamically precedes H3K27me3 [62]. Initial Polycomb deposition occurs across the entire chromosome, but is most prevalent in intergenic regions, consistent with the observation that PCGF3/5-PRC1 normally targets chromatin pervasively to generate low level H2AK119ub1 deposition genome-wide [7,46^•^,^62^]. Further biochemistry [58^••^], superresolution microscopy [57^••^], and proteomics experiments [47,60] have also confirmed a close association between PCGF3/5-PRC1 complexes and hnRNPK/Xist.

Recent experiments in PRC1 (RING1A/B) knockout fibroblasts, in which XCI is already established, show a reduction but not erasure of H3K27me3 domains [59^•^]. This observation may appear contradictory to the model; however, can plausibly be explained by PRC2 feedback and the propagation of H3K27me3 through cell divisions, both of which are well documented [63, 64, 65]. Likewise, the marginal accumulation of H3K27me3 over gene promoters visible by ChIP-seq after Xist induction in PRC1 or B/C-repeat mutants [46^•^,^60^] can be attributed to increased PRC2 activity in genomic regions where it is naturally targeted (i.e. CpG islands), facilitated by transcriptional silencing or the removal of active chromatin modifications at these sites [66].

From RNA-sequencing it has also become clear that in cellular models of Xist-induced gene repression the functional effect of Polycomb removal is significant but not absolute (Table 1) [46^•^,57^••^,58^••^,59^•^,^60^]. Notably, in initial silencing PRC1 appears more important than PRC2 [46^•^], whose role may be limited to later stages of XCI in differentiation or extraembryonic lineages [67]. A recent study has shown that PRC1/H2AK119ub1 is required for the recruitment of the chromosomal protein SMCHD1, which may account, at least in part for its activity in gene silencing [68].

It is well-documented that initial gain of Polycomb modifications correlates with Xist spreading over the Xi [52,69], and occurs predominantly in intergenic domains [46^•^,^62^] or near CpG islands [70^••^] pre-marked by Polycomb. An emerging concept is that in addition to Xist recruiting Polycomb, Polycomb may also play a role in targeting Xist RNA to sites on chromatin, in conjunction with other known localisation/anchoring factors [71]. One report observed defective Xist chromatin association and spreading upon knockout of *Ring1A/B*, *Eed* or deletion of Xist B-repeat region [59^•^]. These observations fit with other proposed models for Xist and imprinted lncRNAs [70^••^,^72^], although further studies will be important to unveil the mechanism by which Polycomb contributes to lncRNA spreading over chromatin.

The emergence of the new model for recruitment of Polycomb by Xist RNA has led to silencing mechanisms downstream of other lncRNAs previously reported to functionally interact with PRC2 being revisited. One recent example profiled accumulation of Polycomb over Mb-long genomic imprinted regions controlled *in cis* by two lncRNAs paternally expressed in trophoblast stem cells, Airn and Kncq1ot1 [70^••^]. Of note, hnRNPK strongly binds both RNAs, and hnRNPK knockdown significantly reduced Polycomb accumulation over its targets [70^••^], implying that the mechanism of recruitment characterised for Xist may be generalisable to other lncRNAs.

## Conclusion

In recent years the model for Polycomb recruitment by RNA has been overturned, with roles for PRC1 and hnRNPK emerging as key determinants. Consideration of this mechanism may be useful for redefining other previously reported functional interactions between lncRNAs and Polycomb. One such example is HOTAIR, where a recent study which tethered HOTAIR to a reporter found that PRC2 deletion had no effect on its ability to mediate transcriptional silencing [73]. However, PRC1 has not been similarly tested.

Although RNA does not seem to directly recruit PRC2, there is evidence to support a function for RNA in regulating PRC2 activity. Future work will need to establish how these RNA-associated mechanisms are integrated with others, such as H2AK119ub1 and DNA binding, to establish appropriate Polycomb repression during development.

## Conflict of interest statement

Nothing declared.

## References and recommended reading

Papers of particular interest, published within the period of review, have been highlighted as

• of special interest
•• of outstanding interest
